# The Spliced Leader RNA Silencing (SLS) Pathway in Trypanosoma brucei Is Induced by Perturbations of Endoplasmic Reticulum, Golgi Complex, or Mitochondrial Protein Factors: Functional Analysis of SLS-Inducing Kinase PK3

**DOI:** 10.1128/mBio.02602-21

**Published:** 2021-11-30

**Authors:** Uthman Okalang, Bar Mualem Bar-Ner, K. Shanmugha Rajan, Nehemya Friedman, Saurav Aryal, Katarina Egarmina, Ronen Hope, Netaly Khazanov, Hanoch Senderowitz, Assaf Alon, Deborah Fass, Shulamit Michaeli

**Affiliations:** a The Mina and Everard Goodman Faculty of Life Sciences and Advanced Materials and Nanotechnology Institute, Bar-Ilan University, Ramat-Gan, Israel; b Department of Biochemistry and Molecular Biology, Busitema University, Mbale, Uganda; c Chemistry Department, Bar-Ilan University, Ramat-Gan, Israel; d Department of Structural Biology, Weizmann Institute of Science, Rehovot, Israel; University of Georgia

**Keywords:** spliced leader RNA silencing, SLS, trypanosomes, BiP, calreticulin, ERO1, QSOX, TIMRHOM1, PK3-PERK homologue

## Abstract

In the parasite Trypanosoma brucei, the causative agent of human African sleeping sickness, all mRNAs are *trans*-spliced to generate a common 5′ exon derived from the spliced leader (SL) RNA. Perturbations of protein translocation across the endoplasmic reticulum (ER) induce the spliced leader RNA silencing (SLS) pathway. SLS activation is mediated by a serine-threonine kinase, PK3, which translocates from the cytosolic face of the ER to the nucleus, where it phosphorylates the TATA-binding protein TRF4, leading to the shutoff of SL RNA transcription, followed by induction of programmed cell death. Here, we demonstrate that SLS is also induced by depletion of the essential ER-resident chaperones BiP and calreticulin, ER oxidoreductin 1 (ERO1), and the Golgi complex-localized quiescin sulfhydryl oxidase (QSOX). Most strikingly, silencing of Rhomboid-like 1 (TIMRHOM1), involved in mitochondrial protein import, also induces SLS. The PK3 kinase, which integrates SLS signals, is modified by phosphorylation on multiple sites. To determine which of the phosphorylation events activate PK3, several individual mutations or their combination were generated. These mutations failed to completely eliminate the phosphorylation or translocation of the kinase to the nucleus. The structures of PK3 kinase and its ATP binding domain were therefore modeled. A conserved phenylalanine at position 771 was proposed to interact with ATP, and the PK3^F771L^ mutation completely eliminated phosphorylation under SLS, suggesting that the activation involves most if not all of the phosphorylation sites. The study suggests that the SLS occurs broadly in response to failures in protein sorting, folding, or modification across multiple compartments.

## INTRODUCTION

Trypanosoma brucei is a digenetic parasite that cycles between the tsetse fly and a mammalian host. Trypanosomatids are known for their nonconventional gene expression mechanisms, such as trans-splicing (1) and mitochondrial RNA editing (2). Trypanosomal mRNAs undergo *trans*-splicing, whereby a small exon, the spliced leader (SL), provided by the small SL RNA, is donated to all pre-mRNAs by *trans*-splicing (1).

Conventional regulation of protein coding genes based on transcription from defined promoters is absent in trypanosomes, and regulation of gene expression is mostly posttranscriptional (3). It was consequently expected that these parasites would lack a typical unfolded protein response (UPR) mechanism, which in other eukaryotes involves transcriptional activation (4–6). Trypanosomes instead cope with endoplasmic reticulum (ER) stress, such as that induced by treatment with a reducing agent, by preferential stabilization of mRNAs that are essential for their ability to withstand such stress (7).

Under severe stress induced by silencing of mRNA encoding proteins involved in protein translocation into the ER lumen, such as the signal-recognition particle (SRP) receptor (*srα*), *sec61*, the translocation channel, and *sec63*, a factor involved in translocation to the ER, the spliced leader RNA silencing (SLS) pathway is induced (8, 9). SLS has two hallmarks: the reduced abundance of SL RNA and an increased level of SNAP2, an SL RNA specific transcription factor that fails to bind to the SL RNA gene promoter and spreads throughout the nucleus (7, 9). We subsequently showed that many of the factors involved in the preinitiation complex of SL RNA behave like SNAP2 (10). SLS leads to programmed cell death (PCD) (7). Based on these data, we proposed that SLS triggers a unique death pathway similar to the classical caspase-mediated PCD observed in higher eukaryotes (4–6).

The mechanism underlying SLS was revealed by purifying the SL RNA transcription complex under SLS. It was found that the TATA-binding protein TRF4 undergoes phosphorylation on serine 35 by serine-threonine kinase, which was purified with the SL RNA transcription complex, and we termed it PK3. PK3 translocates from the face of the ER to the nucleus during SLS. PK3 is responsible for the activation of the PCD induced by SLS (10).

It is currently unknown if SLS is activated only by perturbations on the ER membrane or whether it is induced by depletion of factors involved in protein modification upon translocation into the ER. A number of conserved factors contribute to the three main modifications of proteins as they enter the ER: folding, disulfide bond formation, and glycosylation. The major ER chaperones that assist in protein folding are calnexin, calreticulin (CRT), and BiP (immunoglobulin heavy-chain binding protein) (11). Unlike calnexin and calreticulin, which monitor both N-linked glycans and unfolded regions on nascent polypeptide chains (12), BiP detects only the latter and is the major contributor to folding of nonglycosylated proteins (13). Trypanosomes like other eukaryotes possess the ER chaperones BiP and CRT (14).

To accomplish disulfide bond formation, protein disulfide isomerase (PDI) oxidizes client proteins, and endoplasmic reticulum oxidoreductin 1 (ERO1) transfers electrons from the reduced PDI to a terminal acceptor, which is usually molecular oxygen and is reduced to H_2_O_2_. ERO1 function is essential for disulfide bond formation in yeast (15). As the oxidative activity of ERO1 is related to the production of H_2_O_2_ and burdens cells with potentially toxic reactive oxygen species (ROS), deregulated ERO1 activity is likely to impair cell fitness (15).

Many proteins that are translocated to the ER undergo glycosylation, which is used both for its effect on protein stability and solubility and as a code to monitor protein folding by ER quality control (ERQC). If a newly synthesized protein folds properly and passes the scrutiny of the ERQC machinery, it can be trafficked beyond the ER. Should the protein fail this inspection, it is targeted for proteasomal degradation in the cytosol via ER-associated degradation (ERAD) (16). In most eukaryotes, the glycan added to proteins in the ER is Glc_3_Man_9_GlcNAc_2_, but in trypanosomes, it is Glc_1_Man_9_GlcNAc_2_ (17). In the folding cycle, glucose is removed from this glycan by glycosidases. Trypanosomes encode only a single glucosidase II (GLU2) (18). If the first round of folding fails, another cycle of folding is initiated by adding back the glucose via the enzyme UDP-glucose: glycoprotein glucosyltransferase (UGGT) (19). When misfolded proteins accumulate in the ER, and to avoid possible aggregation, another enzyme, α1,2-mannosidase, removes a mannose residue to produce Man_8_GlcNac_2_, which is then recognized by the protein ER degradation-enhancing alpha mannosidase (EDEM) (20), and the protein is tunneled to ERAD (21). In cases when the protein is properly folded, the protein moves out of the ER to the Golgi complex through the tubulovesicular membrane clusters of the ER-Golgi intermediate compartment (ERGIC). The ERGIC clusters are mobile transport complexes that deliver secretory cargo from ER exit sites to the Golgi complex. The ERGIC also contributes to the concentration, folding, and quality control of newly synthesized proteins (22).

Trypanosomes cycle between insect and mammalian hosts. In the insect host, the primary surface protein is the procyclin EP, which covers the cell surface of the procyclic form (PCF) of parasite, whereas in the bloodstream form (BSF), the main surface protein is the variant surface glycoprotein (VSG), which undergoes antigenic variation (23). In BSF, protein translocation across the ER is very active because of the massive production of VSG (24, 25). RNA interference (RNAi) silencing of *bip*, *crt*, *glu2*, and *uggt* mRNAs in BSF affected growth and resulted in a swollen ER (26).

A recent proteomics analysis of SLS identified a dramatic increase in the level of mitochondrial Rhomboid-like 1 (TIMRHOM1) (27). Recent studies suggest that this protein is involved in protein translocation to the mitochondria and may be a functional homologue of the mitochondrial pore, TIM23 (28). In eukaryotes, mitochondria are connected to the ER, and homeostasis is maintained between these two cellular domains. In yeast, this contact is mediated by the endoplasmic reticulum (ER)-mitochondrial encounter structure (ERMES) complex (29), and in mammals, this contact involves the voltage-dependent anion selective channel protein (VDAC1), which interacts with ER Ca^2+^ channel IP_3_R (30). In trypanosomes, it is not known which proteins make the contact between these compartments.

In this study, we show that SLS is induced when perturbation in protein sorting or modification is elicited by depletion of factors located not only on the ER membrane but also inside the ER lumen. Silencing of *bip*, *crt*, and *ero1* mRNAs and a Golgi complex-localized oxidoreductase (*qsox*) mRNA in PCF each elicited SLS, as demonstrated by reduction in the level of SL RNA, accumulation of TRF4 in the nucleus, and TRF4 phosphorylation. However, depletion of UGGT, GLU2, EDEM, and ERGIC involved in protein trafficking did not elicit SLS, suggesting that not all the factors present in the ER are essential for protein homeostasis in PCF. In addition, we found that perturbation of proteins outside the ER can induce SLS, such as depletion of the mitochondrial TIMRHOM1. We further show that PK3, the kinase activated to initiate SLS, undergoes phosphorylation on multiple sites during SLS activation. The sites were mapped by mass spectrometry (MS) and verified by mutagenesis. Mutations introduced in S606, -628, -707, and -708 and the combination of four mutations, including S606, -628, -707, and -708, did not completely abolish the phosphorylation and translocation of PK3 to the nucleus. Modeling of the PK3 kinase domain (KD) based on the homologous mammalian PERK allowed us to identify the ATP binding domain of PK3. Mutation of PK3^F771L^ in the ATP binding domain completely abolished the phosphorylation under SLS induction, further demonstrating that PK3 phosphorylation is essential for its translocation to the nucleus. Thus, this study provides evidence that SLS is activated under stress originating from inactivation of functions involved in protein sorting, folding, or modification, but not upon functional inactivation of all essential functions in the cell. SLS induction is not restricted to the ER, but encompasses other organelles of the secretory pathway and the mitochondria.

## RESULTS

### Silencing of some, but not all, ER-resident proteins induces SLS.

SLS was previously shown to be induced in T. brucei upon perturbation of functions localized to the ER membrane (8, 9). We subsequently sought to determine whether SLS is induced also by depletion of factors in the ER lumen. To address this question, we silenced key luminal chaperones, namely, the T. brucei orthologs of *bip* and *crt* mRNAs (31, 32). BiP is a heat shock protein 70 (HSP70) chaperone that aids in the translocation and folding of many nascent proteins through cycles of binding and release, whereas CRT is a lectin chaperone of glycoproteins (16).

**FIG 1:**
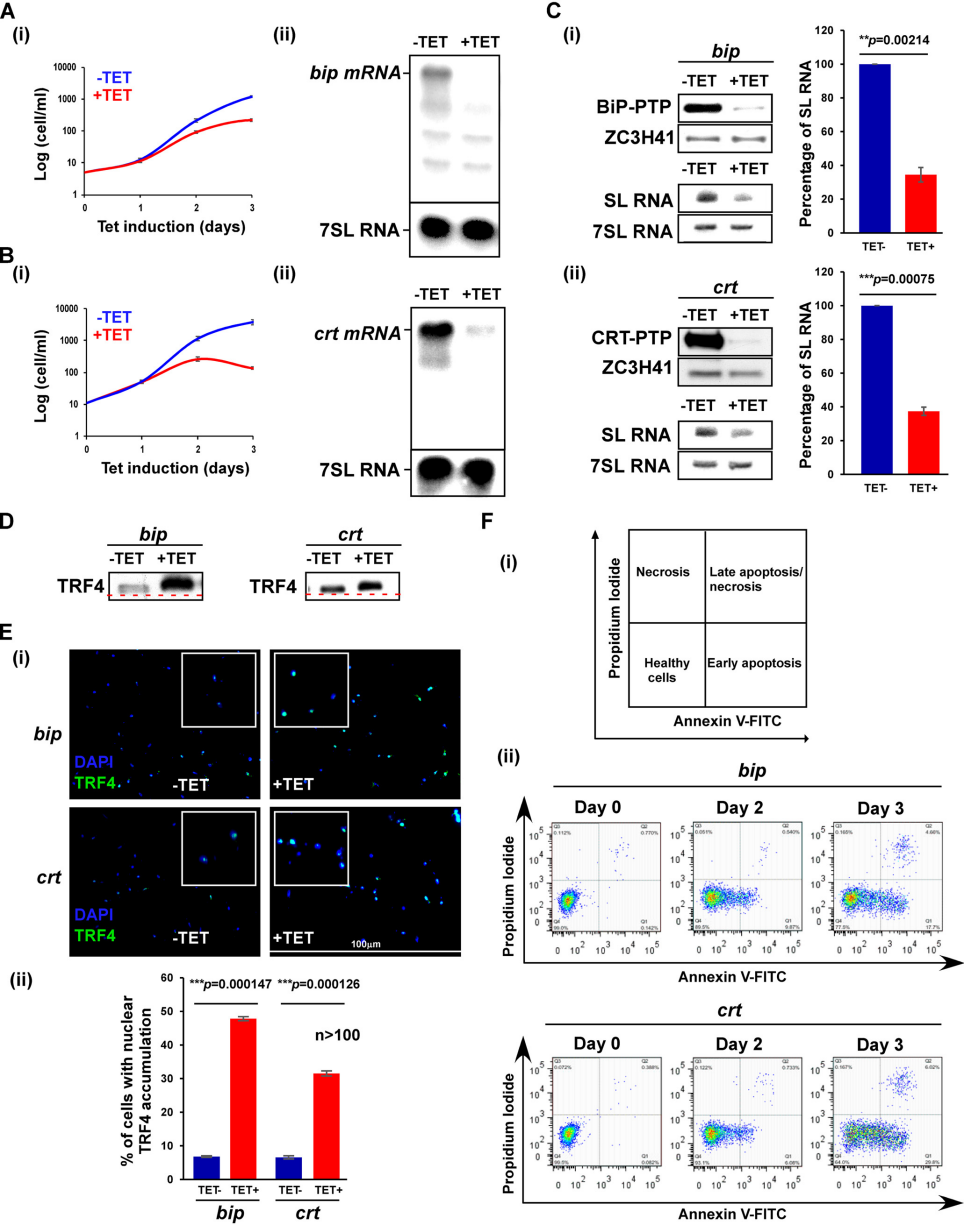
SLS is induced under depletion of factors present in the ER lumen. (A) *bip* mRNA silencing. (i) Growth of cells upon *bip* mRNA silencing. Uninduced cells carrying the silencing construct (−TET) were compared with cells induced for silencing (+TET) at 27°C for the time period indicated. Data are presented as mean ± standard error of the mean (SEM). Experiments were done in triplicate (*n* = 3) using the same clonal population. (ii) Northern analysis. Total RNA (20 μg) was prepared from cells before (−TET) and after (+TET) *bip* mRNA silencing for 2.5 days, separated on a 1.2% agarose–formaldehyde gel, and subjected to Northern analysis using RNA probes, as indicated. 7SL RNA served as loading control. (B) *crt* mRNA silencing was performed in a manner identical to that presented in panel A. (C) The effect of *bip* and *crt* mRNA silencing on SL RNA level. (i, upper panel) Cells carrying the silencing construct for *bip* and PTP-tagged BiP protein were silenced for the 2.5 days, and the entire cell lysate was subjected to Western analysis with the indicated antibodies. The dilutions used for the antibodies are 1:10,000 each for IgG and ZCH341. (Lower panel) Cells carrying the silencing construct for *bip* mRNA were silenced for 2.5 days, and the RNA was subjected to Northern analysis with the indicated antisense probes. Data are presented as mean ± SEM. Experiments were done in triplicate (*n* = 3) using the same clonal population. *P* values were determined by Student's *t* test. (ii) The experiment was performed as in panel i, but using a cell line expressing the *crt* silencing construct. (D) *bip* and *crt* mRNA silencing induces the phosphorylation of TRF4. Nuclear extracts were prepared from cells silenced for *bip* or *crt* mRNA for 2.5 days as previously described (27). The proteins were fractionated on 16% SDS–polyacrylamide gel and subjected to Western analysis with anti-TRF4 antibody (diluted 1:10,000). The line demonstrates a shift in the migration of the protein in uninduced cells compared to the induced population. (E) Immunofluorescence of cells with anti-TRF4 antibody. (i) Cells carrying the silencing constructs were subjected to immunofluorescence assay (IFA) with anti-TRF4 antibody (diluted 1:1,000), which was detected with anti-rabbit IgG (H+L) conjugated to Alexa Fluor 488 (diluted 1:1,000). Cells were visualized by a Nikon eclipse 90i microscope with a Retiga 2000R (QImaging) camera. Enlargements of the nuclear area are shown as insets. (ii) The bar graph represents the quantification of the diffuse pattern of nuclear TRF4 from more than 100 cells per condition. *P* values were determined using Student's *t* test. Data are presented as mean ± SEM. (F) Analysis of exposed phosphatidylserine on the outer membrane of silenced cells. (i) Schematic representation showing the different cell populations detected by staining with annexin V and PI. (ii) Cells silenced for the indicated number of days were reacted with fluorescein isothiocyanate (FITC)-labeled annexin V antibody (MBL) and stained with PI. The identities of the cell lines used are indicated.

To perform silencing of the mRNA, cells expressing T7 opposing and stem-loop RNAi constructs were generated. The cells also expressed PTP-tagged proteins integrated at the authentic loci to monitor the degree of silencing (33). All experiments were performed on cloned populations obtained after transfection. Silencing of *bip* and *crt* mRNAs severely inhibited growth (Fig. 1A and B, panels i). The silencing was verified by Northern analysis (Fig. 1A and B, panels ii) and by observation of the elimination of the PTP-tagged protein after 2.5 days of silencing (Fig. 1C). Next, the level of SL RNA was examined, and clear and significant reduction of the RNA (∼70%) was observed (Fig. 1C, panels i and ii).

SLS is initiated by PK3 phosphorylation, leading to its translocation to the nucleus. The phosphorylation of TRF4 then leads to the dissociation of preinitiation complex and the diffusion of TRF4 in the nucleus (10). To examine the localization of TRF4 and its phosphorylation upon silencing of *bip* and *crt* mRNAs, we used antibodies raised against T. brucei TRF4. The results clearly demonstrate a shift in the migration of TRF4 upon silencing (Fig. 1D). It was previously demonstrated that this shift results from phosphorylation of TRF4 Ser35 (10). Furthermore, a diffuse localization of TRF4 in the nucleus was also observed in the silenced cells (Fig. 1E, panels i and ii). The data presented were based on imaging of more than 100 cells. The results indicate that as a result of silencing, between 30 and 45% of the cells showed diffuse accumulation of TRF4 in the nucleus, and a significant difference was observed between uninduced and induced cells (Fig. 1E, panel ii). Finally, PCD induced by silencing of *bip* and *crt* mRNAs was examined using the annexin V/propidium iodide (PI) method. Induction of apoptosis causes externalization of phosphatidylserine (PS) on the surface of the apoptotic cells. Annexin V binds to the exposed PS of apoptotic cells. Cells were stained with propidium iodide (PI) distinguish between necrotic and apoptotic cells (34) (see schematic presentation in Fig. 1F, panel i). Live cells are not stained by either annexin V or PI (bottom left panel). During early apoptosis, PS is exposed on the surface, but because the plasma membrane is intact, the cells are not stained with PI (bottom right panel). Late apoptotic or necrotic cells lose their membrane integrity and are stained with both PI and annexin V (upper right panel). An early apoptotic population (stained by annexin V) was observed after 2 days of silencing, and a late apoptotic population stained with both annexin V and PI was observed after 3 days of silencing of both factors (Fig. 1F, panel ii), suggesting that, similar to *sec63* mRNA silencing (7), the silencing of *crt* or *bip* mRNAs induced apoptosis.

**FIG 2:**
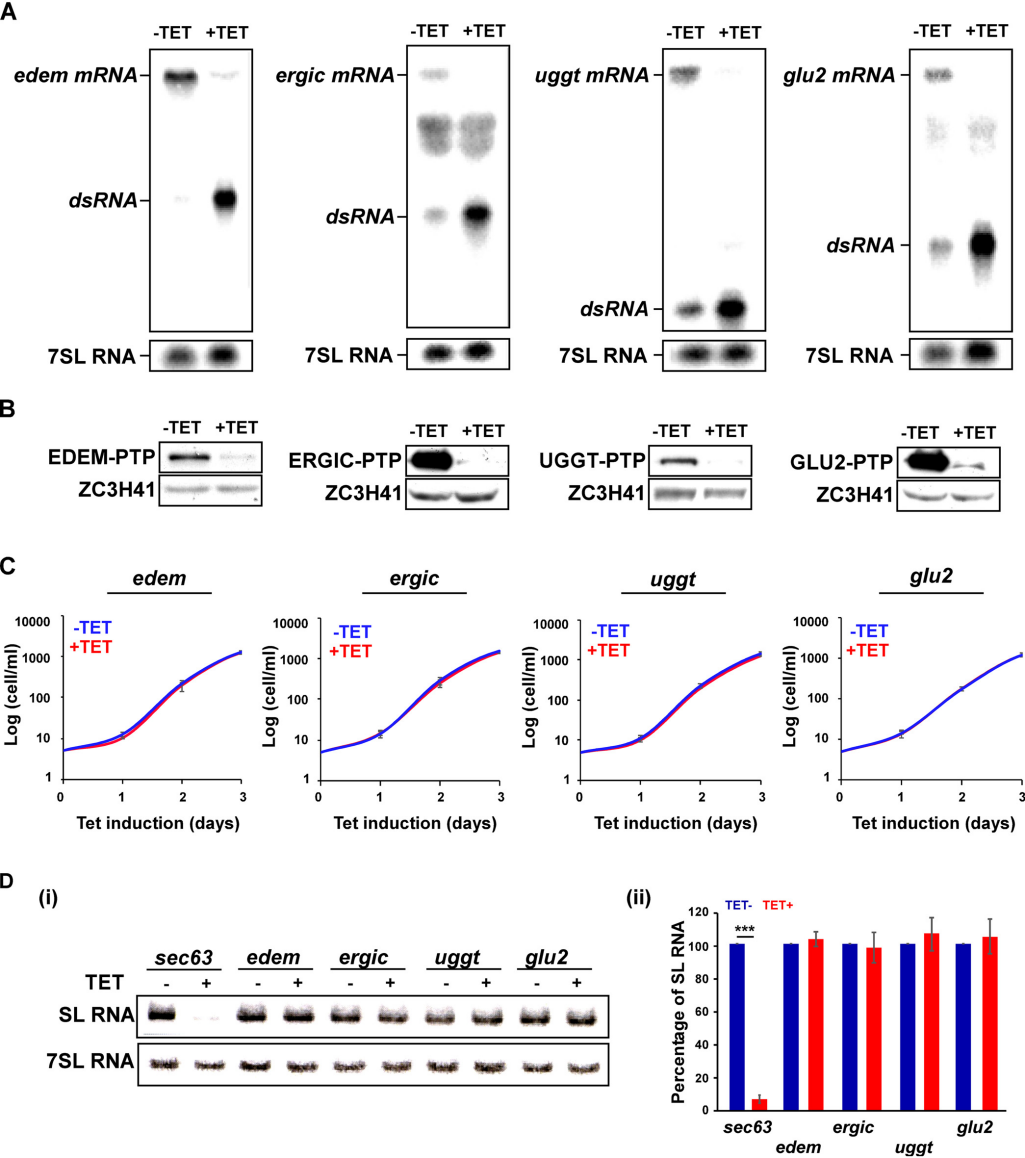
Silencing of *uggt*, *glu2*, *edem*, and *ergic* mRNAs did not affect PCF growth or induce SLS. (A) Northern analysis. Total RNA (20 μg) was prepared from cells before (−TET) and after (+TET) 2.5 days of silencing, separated on a 1.2% agarose–formaldehyde gel, and subjected to Northern analysis using RNA probes, as indicated. 7SL RNA served as loading control. The silenced mRNA and double-stranded RNA (dsRNA) positions are indicated. (B) Western analysis demonstrating the silencing of the mRNA. Western analysis was performed after 2.5 days of silencing demonstrating the depletion of the tagged protein. The dilutions used for the antibodies are 1:10,000 each for IgG and ZCH341. (C) Silencing of the genes has no effect on cell growth. Uninduced cells carrying the silencing construct (−TET) were compared with cells induced for silencing (+TET) at 27°C during the time period indicated. Data are presented as mean ± SEM. Experiments were done in triplicate (*n* = 3) using the same clonal population. (D) SL RNA level upon silencing. (i) Cells carrying the silencing construct for the indicated mRNAs were silenced for 2.5 days, and the RNA was subjected to Northern analysis with the indicated antisense probes. Data are presented as mean ± SEM. Experiments were done in triplicate (*n* = 3) using the same clonal population. *P* values were determined by Student's *t* test. (ii) The bar graph represents the quantification of SL RNA upon silencing of the indicated mRNAs. Data are presented as mean ± SEM. Experiments were done in triplicate (*n* = 3) using the same clonal population. *P* values were determined by Student's *t* test.

After this initial indication that silencing of luminal ER factors mRNAs could induce SLS, we tested whether elimination of additional factors involved in protein folding and quality control in the ER lumen also induces SLS. The factors UGGT, GLU2, and EDEM, which are involved in quality control inside the ER, and ERGIC, which is involved in the transport from the ER to the Golgi complex, were studied. Cells expressing either T7 RNAi or stem-loop constructs and expressing the PTP-tagged proteins were prepared. Silencing was verified by both Northern and Western blot analyses based on the tagged proteins, and efficient silencing was observed (Fig. 2A and B). The silencing did not affect cell growth (Fig. 2C). No difference in the levels of SL RNA between uninduced and silenced cells was observed, in contrast to almost 90% reduction in *sec63* silenced cells (Fig. 2D, panels i and ii), suggesting that these factors are not essential for PCF, as opposed to bloodstream-form trypanosomes (26).

### Depletion of the sulfhydryl oxidases ERO1 and QSOX induces SLS.

**FIG 3:**
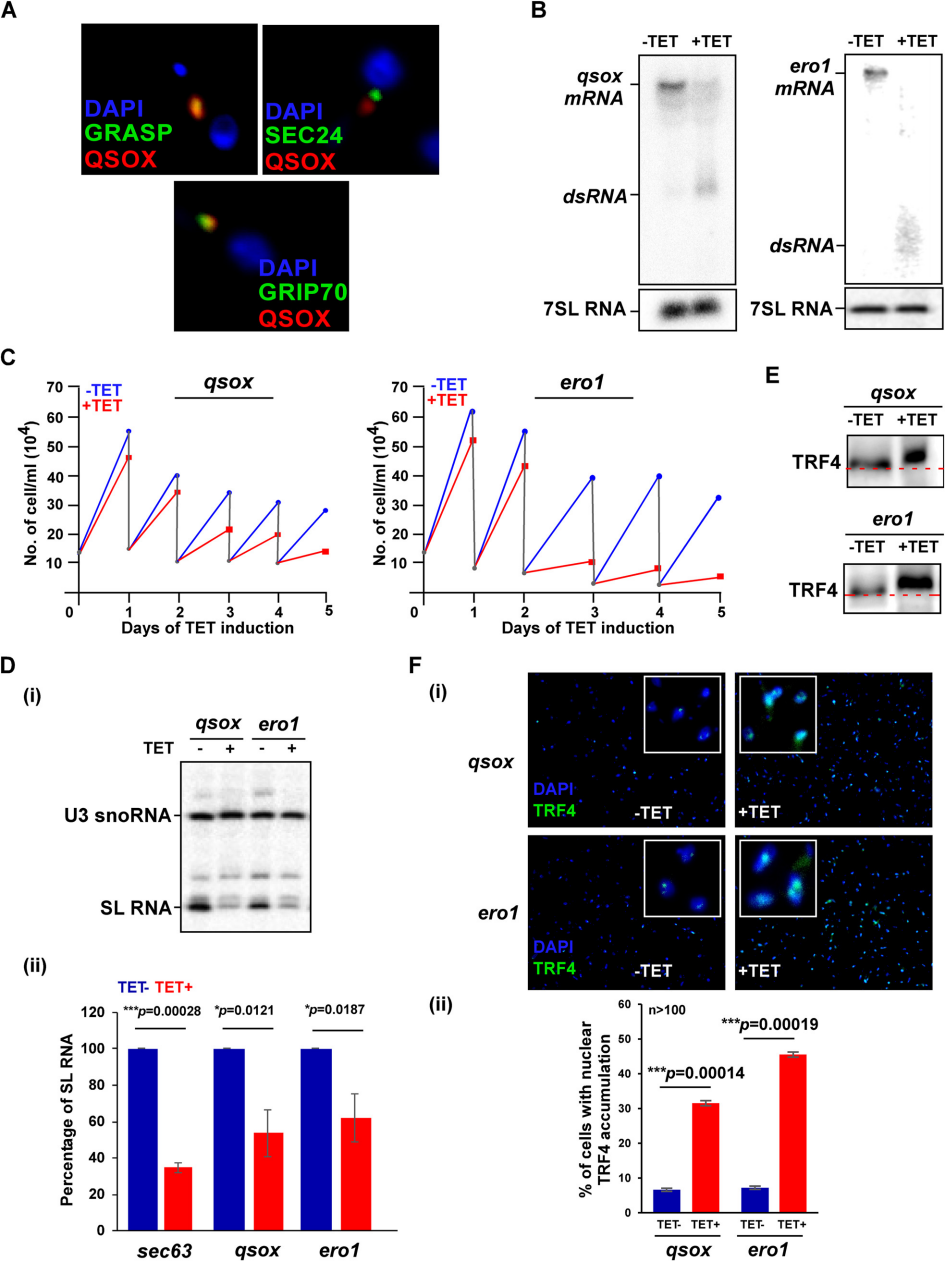
The silencing of *qsox* and *ero1* mRNA induces SLS. (A) Localization of QSOX. PCF cells were subjected to IFA with rat anti-QSOX antibody, which was detected using anti-rat IgG conjugated to Alexa Fluor 488 (diluted 1:10,000) as well as with either rabbit GRASP, GRIP70, or SEC24 antibody (36, 37) (diluted 1:1,000), and detected with anti-rabbit IgG conjugated to Cy3. Cells were visualized by the Nikon eclipse 90i microscope with a Retiga 2000R (QImaging) camera. Nuclei were stained with DAPI (4′,6-diamidino-2-phenylindole). (B) Northern analysis. Cells expressing the stem-loop silencing constructs for *qsox* and *ero1* mRNA were induced for 2.5 days, and RNA was subjected to Northern analysis with their gene-specific RNA probes. The positions of *qsox* and *ero1* mRNA and dsRNA are indicated. (C) *qsox* and *ero1* silencing induces growth arrest. The growth of cells prior to and after tetracycline addition was compared. Both uninduced and induced cultures were diluted daily to 2 × 10^4^ cells per ml. (D) The effect of *qsox* and *ero1* mRNA silencing on SL RNA. (i) Total RNA (10 μg) was prepared from *qsox* and *ero1* mRNA silenced cells after 2.5 days of induction and was subjected to primer extension with antisense SL RNA oligonucleotide. The level of U3 was used to control for equal loading. The samples were fractionated on 6% polyacrylamide–7 M urea gels. (ii) The bar graph represents the quantification of SL RNA upon silencing of the indicated mRNAs. Data are presented as mean ± SEM. Experiments were done in triplicate (*n* = 3) using the same clonal population. *P* values were determined by Student's *t* test. (E) *qsox* and *ero1* mRNA silencing induces the phosphorylation of TRF4. Nuclear extracts were prepared from cells after 2.5 days of silencing, as previously described (27). The proteins were fractionated on 16% SDS–polyacrylamide gel and subjected to Western analysis with anti-TRF4 antibody (diluted 1:10,000). The line demonstrates a shift of protein migration in uninduced cells compared to the induced population. (F) Immunofluorescence of QSOX and ERO1 cells with TRF4 antibody. (i) Cells carrying the silencing constructs either uninduced or after 2.5 days of silencing were subjected to IFA with TRF4 antibody (diluted 1:1,000), which was detected with anti-rabbit IgG (H+L) conjugated to Alexa Fluor 488 (diluted 1:1,000). The nucleus was stained with DAPI. Cells were visualized under the Nikon eclipse 90i microscope with a Retiga 2000R (QImaging) camera. Enlargements of the nuclear area are shown as insets. (ii) The bar graph represents the quantification of diffuse nuclear TRF4 from more than 100 cells per condition. *P* values were determined by Student's *t* test. Data are presented as mean ± SEM.

We next asked whether interfering with secretory pathway functions other than chaperoning and quality control could induce SLS. Another feature of the secretory pathway is its ability to promote formation of disulfide bonds during protein folding and assembly. Mammalian ERO1 catalyzes disulfide bond formation in the ER. Another catalyst of disulfide formation, QSOX, is localized to the Golgi apparatus or secreted from mammalian cells (35). Antibodies raised against T. brucei QSOX were used to establish the localization of this enzyme in the parasite. Colocalization of QSOX was examined with the *trans*-Golgi network (TGN) protein marker GRIP70 (36), the Golgi stack marker GRASP (37), and SEC24, which marks the budding of COPII vesicles from the ER (36). The results in Fig. 3A show the closest colocalization of T. brucei QSOX with GRASP, indicating that QSOX is Golgi complex localized in T. brucei as in mammalian cells.

Silencing of either *qsox* or the *ero1* sulfhydryl oxidase mRNAs (Fig. 3B) indicates that these factors are essential for growth (Fig. 3C). The level of SL RNA was examined by primer extension, and reduction of about 50% of SL RNA was observed (Fig. 3D, panels i and ii). Primer extension analysis was used to demonstrate that SL RNA is not degraded under SLS, suggesting that the reduction in SL RNA level stems from SL RNA transcription shutoff. The phosphorylation of TRF4 (Fig. 3E) demonstrates retardation of TRF4 upon silencing. Next, the distribution of TRF4 in the nucleus was examined upon silencing. Silencing resulted in diffuse accumulation of TRF4 in the nucleus compared to uninduced cells (Fig. 3F, panels i and ii). These results show that depletion not only of factors localized on the ER, but also those in the ER lumen as well as outside the ER, can induce SLS.

### Depletion of TIMRHOM1 affects protein translocation across the ER and mitochondrial function and induces SLS.

**FIG 4:**
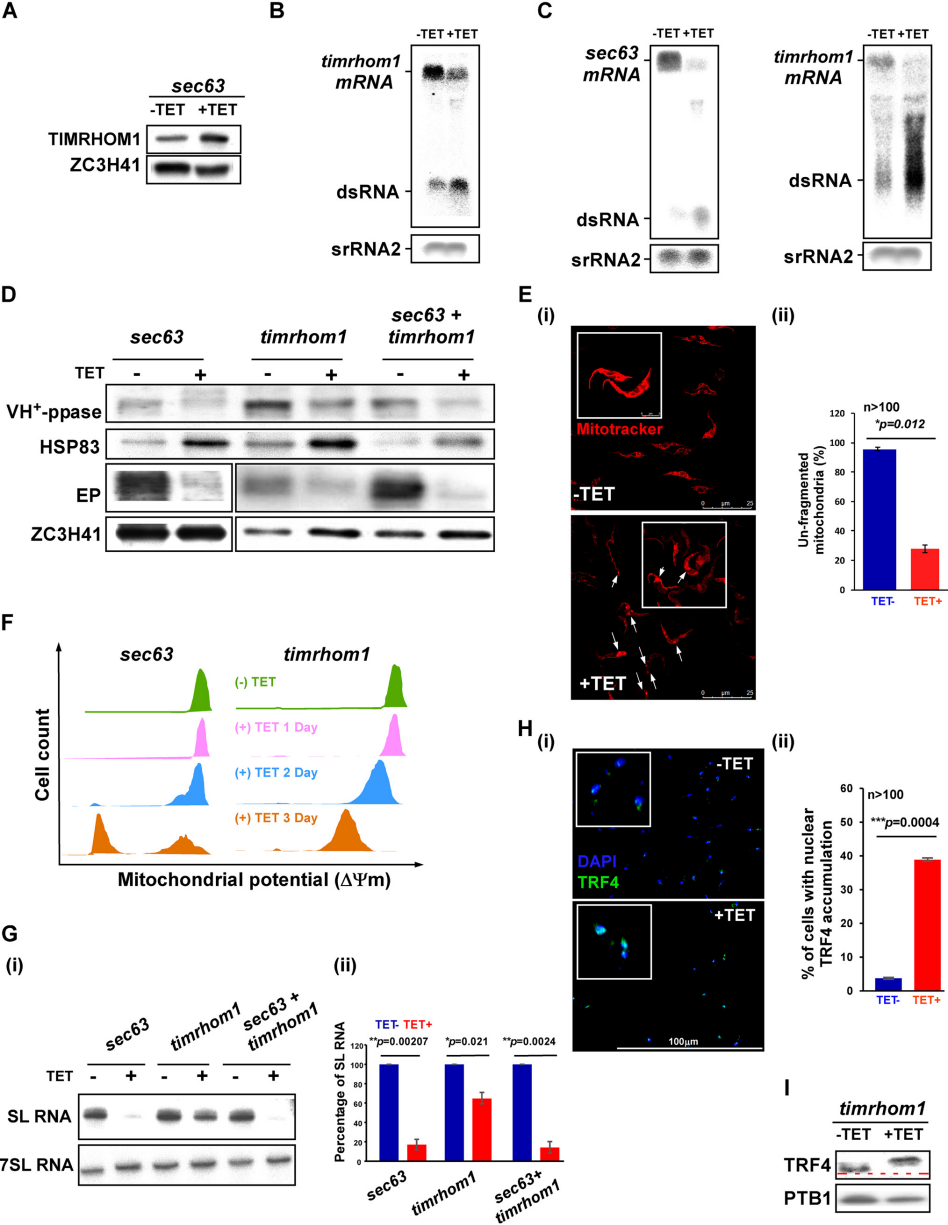
Silencing of *timrhom1* mRNA induces SLS. (A) The level of TIMRHOM1 upon SLS induction. Cells carrying the silencing construct for *sec63* mRNA were silenced for 2.5 days, and the whole-cell lysate was subjected to Western analysis with the indicated antibodies. The dilutions used for the antibodies were 1:500 for TIMRHOM1 and 1:10,000 for ZCH341. (B) Silencing of *timrhom1* mRNA. Cells expressing the stem-loop construct for silencing *timrhom1* mRNA were induced for 2.5 days, RNA was subjected to Northern analysis with the gene-specific RNA probes, and srRNA2 was used to control for equal loading. The positions of the mRNA and dsRNA bands are indicated. (C) Cosilencing of *sec63* and *timrhom1* mRNAs. Northern blot analysis was performed on RNA from cells carrying the silencing constructs for both *sec63* and *timrhom1* mRNAs. The blots were probed separately with gene-specific probes, and srRNA2 was used to control for equal loading. (D) Effect of *timrhom1* mRNA silencing on protein translocation across the ER. Proteins from *sec63*, *timrhom1*, and *sec63 timrhom1* mRNA cells silenced for 2.5 days were subjected to Western analysis and reacted with antibodies to antivacuolar VH^+^-ppase antiserum (diluted 1:5,000) as well as anti-EP monoclonal antibody (Mab) 247 (diluted 1:10,000). (E) Silencing of *timrhom1* mRNA induces mitochondrial fragmentation. (i) Cells expressing the silencing construct for *timrhom1* mRNA were silenced for 2.5 days and stained with MitoTracker. Enlargements of cells are shown in the insets. Cells with fragmented mitochondria are marked with white arrows. (ii) The bar graph represents the quantification of unfragmented mitochondria from more than 100 cells per condition. *P* values were determined by Student's *t* test. Data are presented as mean ± SEM. (F) Silencing of *timrhom1* mRNA induces loss of mitochondrial membrane potential. Cells were harvested and loaded with 150 nM TMRM in serum-free medium. The samples were incubated in the dark for 15 min at 27°C and then analyzed by FACS. The cell count of cells along with ΔΨm is plotted comparing the cells before and during the first 3 days of silencing. The data compare *sec63* silenced cells to *timrhom1* mRNA silencing. (G) *timrhom1* mRNA silencing reduces the level of SL RNA. (i) Total RNA (10 μg) was prepared from *sec63*, *timrhom1*, and *sec63 timrhom1* silenced cells after 2.5 days of induction, fractionated on 10% polyacrylamide–7 M urea gel, and subjected to Northern analysis with SL RNA and 7SL RNA probes. (ii) The bar graph represents the quantification of SL RNA upon silencing of the indicated mRNAs. Data are presented as mean ± SEM. Experiments were done in triplicate (*n* = 3) using the same clonal population. *P* values were determined using Student's *t* test. (H) Changes in TRF4 localization upon *timrhom1* mRNA silencing. (i) Cells carrying the *timrhom1* mRNA silencing construct were induced for 2.5 days and subjected to IFA with TRF4 antibody (diluted 1:1,000), which was detected with anti-rabbit IgG (H+L) conjugated to Alexa Fluor 488 (diluted 1:1,000). The nucleus was stained with DAPI. Cells were visualized by the Nikon eclipse 90i microscope with a Retiga 2000R (QImaging) camera. Enlargements of the nuclear area are shown as insets. (ii) The bar graph represents the quantification of diffused pattern of nuclear TRF4 upon silencing from more than 100 cells per condition. *P* values were determined by Student's *t* test. Data are presented as mean ± SEM. (I) *timrhom1* mRNA silencing induces the phosphorylation of TRF4. Nuclear extracts were prepared from cells after 2.5 days of silencing, as previously described (27). The proteins were fractionated on 16% SDS–polyacrylamide gel and subjected to Western analysis with anti-TRF4 antibody (diluted 1:10,000). The line demonstrates the shift of protein migration in uninduced cells compared to the induced cells.

The proteome analysis of cells in which SLS had been induced by silencing of *sec63* indicated an increase in the level of TIMRHOM1 protein (27). To verify this elevation, the level of the protein was examined upon SLS induced by *sec63* silencing, and a 4-fold increase was observed (Fig. 4A). Thus, TIMRHOM1 appears to be connected to the SLS pathway. Because of the increase in TIMRHOM1 during *sec63* silencing, we suggested that its induction may assist in coping with protein translocation defects across the ER. To test the effect of depleting TIMRHOM1, a stem-loop construct was used for silencing, which was verified by Northern analysis (Fig. 4B). We also cosilenced *sec63* with *timrhom1* to examine if *timrhom1* mRNA silencing exacerbates the protein translocation defects across the ER. The cosilencing was confirmed by Northern analysis (Fig. 4C). To examine the effect of the cosilencing on protein translocation across the ER, the levels of two proteins, VH^+^-ppase, which is a membrane protein located in the acidocalcisome, and the glycosylphosphatidylinositol (GPI)-anchored protein EP, both affected by *sec63* silencing (8), were examined. To our surprise, since TIMRHOM1 is localized to the mitochondria (28), a clear reduction in the levels of both ER translocated proteins was observed upon *timrhom1* mRNA silencing (Fig. 4D). Cosilencing did not worsen the defects (Fig. 4D). To verify that, indeed, TIMRHOM1 affects mitochondrial integrity and function, mitochondria were stained with MitoTracker, and mitochondrial function was assessed using tetramethyl rhodamine methyl ester (TMRM), which accumulates in the negatively charged active mitochondria, and reports on mitochondrial membrane potential (ΔΨm). MitoTracker staining showed a single long mitochondrion in uninduced cells, which upon silencing appeared fragmented (indicated by arrows in Fig. 4E, panel i). A significant difference in fragmentation was detected upon silencing (Fig. 4E, panel ii). The reduction in ΔΨm was more rapid in *timrhom1* mRNA silenced cells compared to *sec63* mRNA silenced cells (Fig. 4F), as expected, because TIMRHOM1 is a mitochondrial protein involved in protein translocation and the effect on mitochondria is primary, whereas the effect of *sec63* silencing on the mitochondria is likely to be secondary.

Because *timrhom1* mRNA silencing induced mitochondrial fragmentation, as in SLS induction, we examined whether *timrhom1* mRNA silencing itself induces SLS. Indeed, Northern analysis based on three replicates showed that the level of SL RNA was reduced upon *timrhom1* mRNA silencing, but to a lesser extent compared to *sec63* silencing (Fig. 4G, panels i and ii). Immunofluorescence of TRF4 in the nucleus demonstrates the massive accumulation of TRF4 upon *timrhom1* mRNA silencing (Fig. 4H, panels i and ii). In addition, Western analysis with anti-TRF4 (Fig. 4I) showed that silencing of *timrhom1* mRNA induces the phosphorylation of TRF4. Thus, SLS induction is not restricted to perturbation of only secretory pathway compartments but can also be induced by defects in the mitochondria due to blockage of protein translocation.

### PK3 undergoes phosphorylation under SLS, which affects its translocation to the nucleus.

The serine-threonine family eIF2 kinases such as PERK undergo autophosphorylation upon activation (38–40). PK3 belongs to this family of kinases (41) and hence is expected to undergo autophosphorylation upon activation. The activation of PERK is followed by the autophosphorylation of the kinase domain, which provides PERK with full catalytic activity (42). In the crystal structure of the PERK kinase domain, the N-terminal lobes are responsible for dimerization, forming homodimers constituted by two back-to-back monomers. Following autophosphorylation at the activation loop (C-lobe), the structure becomes ordered and is partially stabilized and able to bind its substrate (38).

**FIG 5:**
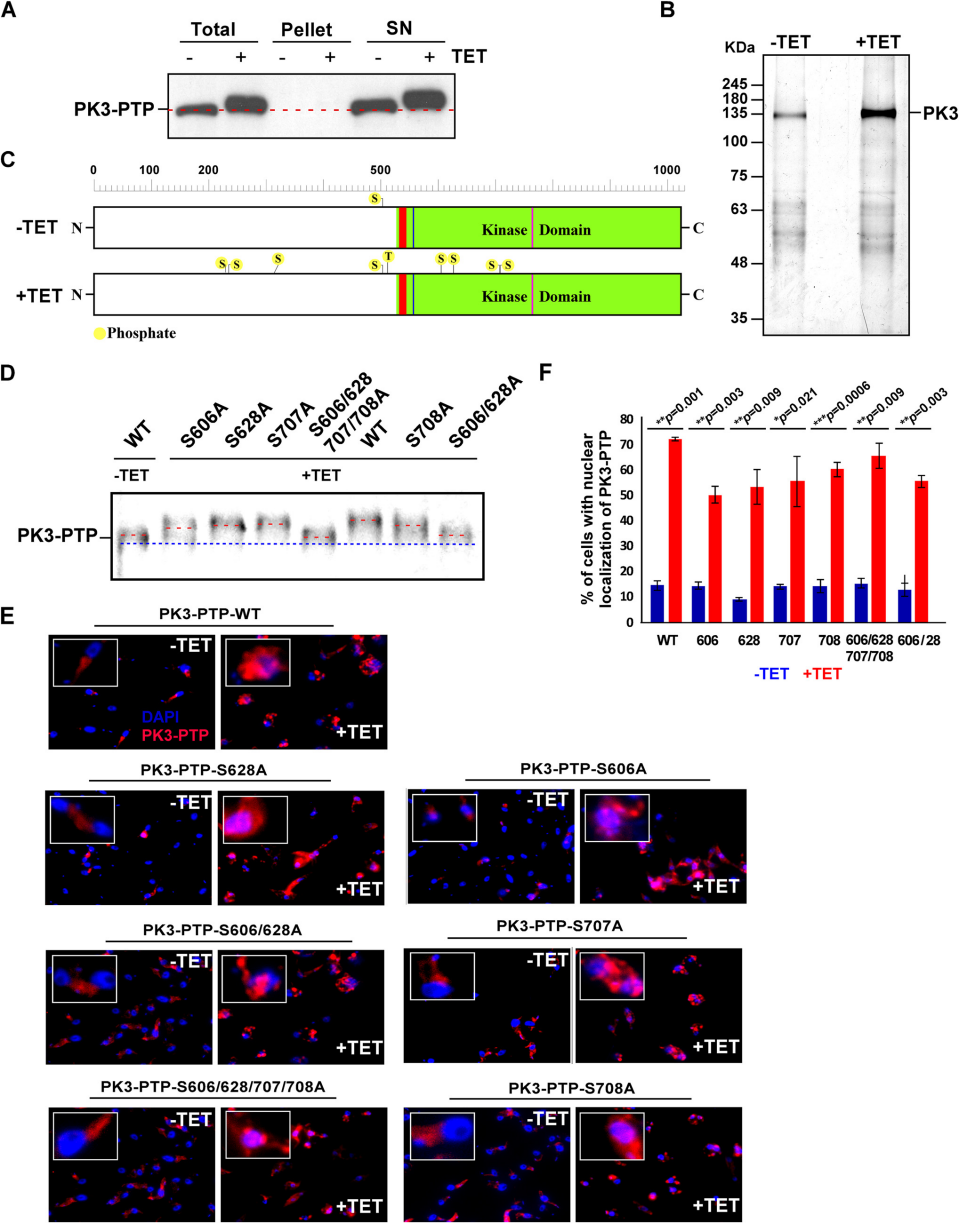
PK3 phosphorylation under SLS. (A) Purification of PK3-PTP protein from SLS-induced cells. Extract was prepared from 10^10^ silenced cells after treatment with detergent-rich buffer as detailed in Materials and Methods. Aliquots from total cell extracts, soluble material (SN), and insoluble material (1/50) were subjected to Western analysis. (B) Purified PK3. The purification was performed as previously described (79) from the extract in panel A above. The eluted material (1/10) was fractionated on a 10% SDS–polyacrylamide gel and stained with silver. The remaining material was fractionated on a gel, eluted, and subjected to MS (Table S2) (C) Schematic presentation of the localization of the sites that undergo phosphorylation under SLS. The kinase domain (positions 528 to 1022) is depicted in green, and the ATP binding domain (positions 534 to 542) is in red. (D) Mutations in the phosphorylation sites and their effect on PK3 modification. The mutations were constructed as described in Materials and Methods. Cells carrying the *sec63* mRNA silencing construct and the different PK3 mutations were subjected to Western analysis with rabbit IgG serum (Sigma) followed by reaction with anti-rabbit IgG (H+L) conjugated to horseradish peroxidase. The positions of the mutations are indicated, and the line shows the difference in migration of the wild-type tagged protein and the PK3 mutants. (E) Immunofluorescence of PK3 upon induction of SLS. Cells expressing the *sec63* silencing construct and PK3-PTP mutations were silenced for 2.5 days. The cells were subjected to IFA by first reacting the cells with rabbit IgG serum (Sigma) (diluted 1:1,000) and then with anti-rabbit IgG (H+L) conjugated to Alexa Fluor 488 (diluted 1:1,000). The nucleus was stained with DAPI. Enlargements of the nuclear area are shown as insets. (F) The bar graph represents the quantification of nuclear localization of PK3-PTP upon *sec63* silencing from more than 100 cells per condition. *P* values were determined by Student's *t* test. Data are presented as mean ± SEM. The identity of each mutation is indicated.

We have so far shown that different cues induce SLS, including low pH, chemicals such as dithiothreitol (DTT) and 2-deoxy-d-glucose (2-DG), and depletion of factors involved in protein translocation into the ER (7–9), as well as factors involved in protein folding or assembly localized in the ER lumen, or even the Golgi apparatus or mitochondria, as demonstrated here. It is currently unknown whether all of these cues activate PK3 in the same manner. To answer this question, we first identified the protein modifications that PK3 undergoes during SLS induced by *sec63* silencing. To this end, we used the cell line expressing both the *sec63* silencing and tagged PK3-PTP constructs. The purification to extract the majority of the kinase required several detergents, as specified in Materials and Methods, and only under these conditions was it possible to release PK3 from its association with membranes. The PTP-tagged protein was purified as previously described (10) and selected on a titanium oxide column to enrich for phosphorylated proteins. Alternatively, the purified PK3 was separated on 10% SDS–polyacrylamide, purified from the gel, and subjected to mass spectrometry (MS). The detection of PK3 after the detergent extractions and the quality of purification of the PK3 before and after *sec63* silencing are presented in Fig. 5A and B, respectively. The list of the phosphorylation sites revealed by these two purification methods is presented in Table S2 in the supplemental material. The phosphorylation sites induced by SLS on the PK3 sequence are presented in Fig. 5C, which shows that four of the phosphorylation sites are clustered in the kinase domain.

10.1128/mBio.02602-21.4 TABLE S2 PK3 purification and mass spectrometry of the phosphorylated residues. PK3-PTP was affinity purified as described in Materials and Methods and in the legend to Fig. 5. (Part A) Mass spectrometry analysis of proteins purified on titanium oxide column. (Part B) Mass spectrometry of isolated protein. The protein was excised from a 10% SDS–polyacrylamide gel and subjected to MS. The peptide sequences and phosphorylation sites obtained in parts A and B are indicated. PSMs, peptide spectrum matches, which indicate the total number of identified peptide sequences. Download Table S2, XLS file, 0.03 MB. Copyright © 2021 Okalang et al. 2021 Okalang et al. https://creativecommons.org/licenses/by/4.0/ This content is distributed under the terms of the Creative Commons Attribution 4.0 International license.

To verify the MS results and demonstrate that these sites are indeed phosphorylated as a result of SLS induction, single, double, as well as quadruple mutations were introduced at the following positions in PK3: 606, 628, 707, 708, 606 plus 628, and 606 plus 628 plus 707 plus 708. The serine-to-alanine mutant constructs were introduced into cells carrying the *sec63* silencing construct. The results demonstrate that the mutated positions are indeed phosphorylated under SLS, since upon induction of SLS, the mutated tagged protein migrated differently than the wild-type tagged PK3 (Fig. 5D). Retardation of migration is consistent with the number of mutated residues. Only the mutant in which four resides were mutated exhibited a defect in its ability to undergo phosphorylation. Next, we examined whether the mutated proteins could translocate to the nucleus. To this end, we examined the localization of the mutated proteins by immunofluorescence using an antibody that recognizes protein A present in the PTP tag. The results (Fig. 5E and F) demonstrate that translocation to the nucleus of the different mutations was compromised and was less efficient compared to the wild-type PK3. However, since PERK-like kinases function as dimers (38), it is possible that the mutants formed heterodimers composed of wild-type and mutant PK3 monomers, which could still translocate to the nucleus.

**FIG 6:**
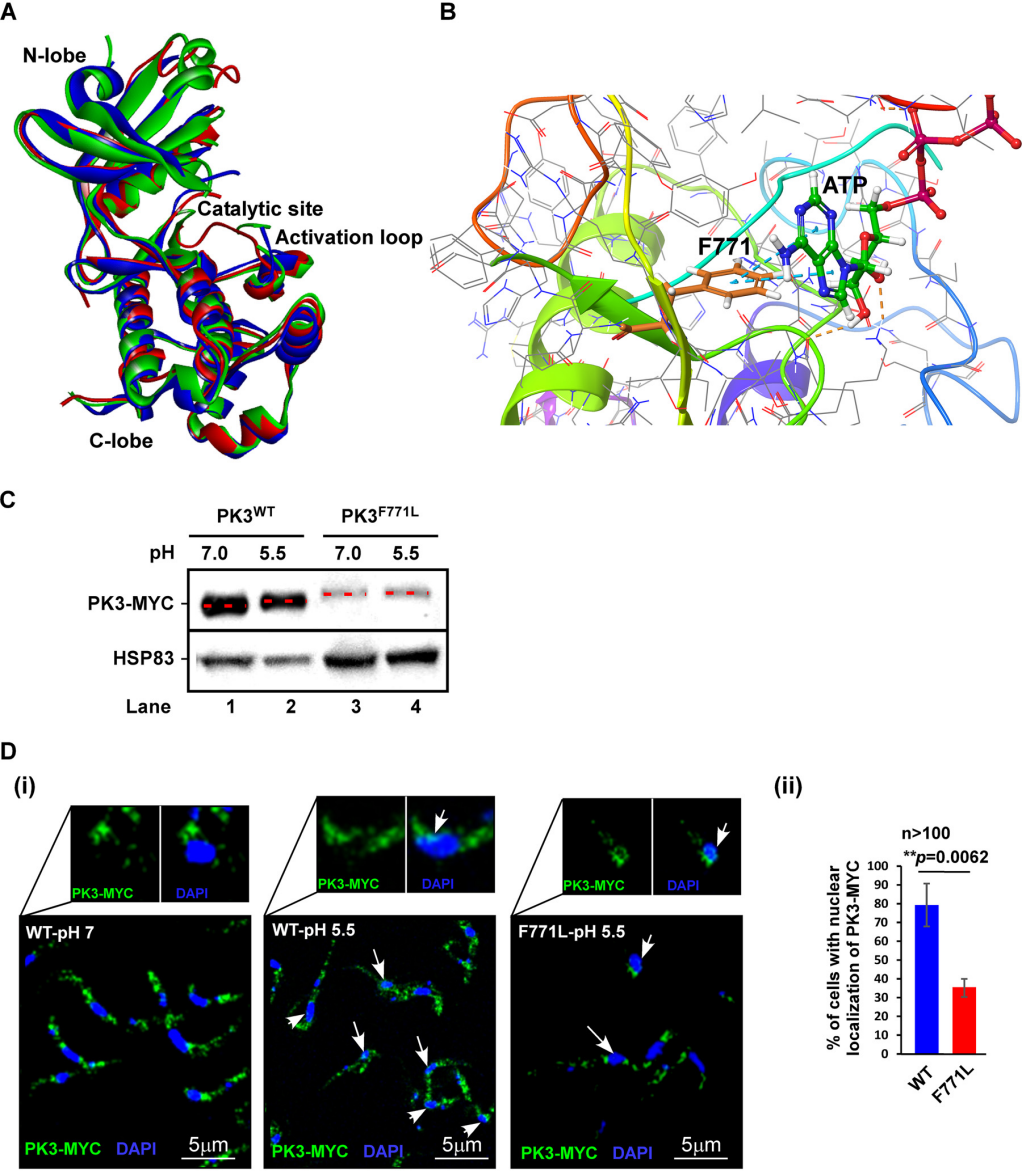
Modeling PK3 KD. (A) Homology model of PK3 KD superimposed on the crystal structures of human and mouse PERK KDs. The modeling was based on the human PERK (43) and mouse PERK (38). The structures are shown as ribbon diagrams and colored in red, green, and blue, representing PK3, human, and mouse proteins, respectively. The positions of the N-lobe, C-lobe, catalytic site, and activation loop are indicated. (B) Docking of ATP in its binding site on the proposed PK3 model. The Phe 771 (F771) that makes contact with the adenosine moiety of ATP via two T-shaped π-π interactions is indicated. The interactions are illustrated with blue dashed lines. (C) PK3^WT^-MYC-tagged protein but not PK3^F771L^ undergoes phosphorylation under SLS. Cells expressing the PK3 tagged with MYC were incubated at either pH 7.0 (control) or pH 5.5 for 3 days to induce SLS as previously described (9). Whole-cell lysates were prepared and subjected to Western analysis with anti-MYC antibody (9E10; Santa-Cruz). HSP83 was used to monitor protein levels. The lanes are numbered. (D) Changes in the localization of PK3-MYC upon SLS induction. (i) Cells were incubated at the indicated pH as described above and subjected to IFA with anti-MYC antibodies. The nuclei were stained with DAPI. Enlargement of a portion of the images is shown in the inset. The cells harboring the tagged PK3-MYC in their nucleus are indicated with arrows. (ii) Bar graph representing the quantification of nuclear localization of PK3-MYC at pH 5.5 from more than 100 cells per condition. *P* values were determined by Student's *t* test. Data are presented as mean ± SEM. The identity of each mutation is indicated.

To examine if complete abrogation of phosphorylation would completely eliminate the nuclear localization under SLS, we performed structural modeling of the PK3 kinase domain (KD), to identify residues that could potentially inactivate the kinase function. The homology model of PK3 (UniProtKB identifier [ID] Q583N6) was generated based on the crystal structures of the homologous proteins: human PERK (PDB ID 4G31) (43) and mouse PERK (PDB ID 3QD2) (38) KDs. Sequence alignment of the entire KDs showed 14.6% sequence identity between PK3 and its homologs (see Fig. S1 in the supplemental material). However, since the template structures mentioned above lack many residues present in PK3, removing the corresponding missing residues from the query sequence of PK3 increased the sequence identity to 26.9%. The homology model for PK3 KD was produced by the MODELLER v.9.2 program (44, 45), and further side-chain refinement was performed using Predict Side Chains tool as implemented in the Maestro program (Schrödinger, USA). The resulting homology model (Fig. 6A) was found to have favorable stereochemical qualities, with 96.3% of residues residing in the most favored regions and additionally allowed regions of the Ramachandran plot and an overall G-factor of −0.14. The model’s Prosa profile was similar to the template, with a Z-score of −4.76 (46). The template/target root mean square deviation (RMSD) of atomic positions was calculated to determine the similarity between the two protein structures. The smaller the RMSD, the more similar the structures. Values based on Cα of the backbone atoms were 2.1 and 2.4 Å for PK3 model/human PERK (43) and PK3 model/mouse PERK (38), respectively (Fig. 6A). All of these parameters support the quality of the proposed model. The model of the PK3 KD (red) is shown in Fig. 6A and demonstrates high similarity to the two templates human PERK (depicted in green) and mouse PERK (depicted in blue). The functional domains are indicated. After optimizing the model of PK3 KD, the ATP binding domain was predicted and was found to have 63.6% identity with the homologs (Fig. S1). The phenylalanine Phe771 (F771) (indicated in Fig. 6B) present in the ATP binding domain was found to be conserved among the sequences used for building the homology model (Fig. 6B) (i.e., Phe943 in 4G31 of human PERK and Phe942 in 3QD2 of mouse PERK). This Phe771 is likely to contribute to the binding of ATP. In addition, the crystal structure of the complex between the PERK inhibitor GSK2606414 and the human PERK protein reveals that the ligand forms a π interaction with Phe943 in the protein’s ATP binding site, supporting the role of this residue in ATP binding (43). Finally, docking using the Glide SP protocol (47, 48) of ATP into the ATP binding site of the PK3 model resulted in two T-shaped π-π interactions between the adenosine moiety of ATP and the conserved Phe771 (Fig. 6B, marked with dashed arrows). Taken together, these observations suggested that mutating Phe771 may prevent PK3 from binding ATP, thereby rendering the protein nonfunctional. To examine if this is indeed the case, a mutation was introduced in this position converting phenylalanine to leucine in the C-terminal MYC-tagged version of the gene. The gene was integrated *in situ* in PCF cells, and induction of SLS was performed by a low pH of 5.5 (9). Upon induction of SLS at low pH, the wild-type MYC-PK3 migration was retarded, suggesting that the MYC-tagged protein was functional (Fig. 6C, lanes 1 and 2). However, the MYC-tagged mutated protein was unable to undergo phosphorylation (Fig. 6C, lanes 3 and 4). Next, we examined the translocation of the protein following SLS induction by low pH (Fig. 6D, panels i and ii). The results show a dramatic effect of this mutation on the ability of the mutated protein to translocate to the nucleus (only 30%) (Fig. 6D, panel ii). The residual translocation could be explained by formation of dimers with a wild-type monomer. However, the marked reduction in the translocation to the nucleus of the nonphosphorylated protein upon conditions that induce SLS suggests that PK3 phosphorylation is essential for its translocation.

10.1128/mBio.02602-21.1 FIG S1 Sequence alignment between T. brucei PK3, human PERK (PDB ID 4G31) (43), and the mouse PERK (PDB ID 3QD2) (38) kinase domain. Removing the residues that are colored in black increases the sequence identity from 14.6% to 26.9%. Focusing on the ATP binding site (boxed in red) further increases the sequence identity to 63.6%. The conserved phenylalanine residues in the binding site of ATP are shown in a black box. Identical, similar, and nonsimilar residues are colored in dark blue, light blue, and white, respectively. Download FIG S1, TIF file, 1.9 MB. Copyright © 2021 Okalang et al. 2021 Okalang et al. https://creativecommons.org/licenses/by/4.0/ This content is distributed under the terms of the Creative Commons Attribution 4.0 International license.

## DISCUSSION

In this study, we demonstrate that SLS is induced by depletion of protein factors, including not only those located in the ER membrane, but also factors inside the ER and involved in protein folding (BiP and CRT) and protein oxidative modification (EROI). SLS was also found to be induced by depletion of an enzyme catalyzing disulfide bond formation located in the Golgi apparatus (QSOX). The most surprising finding was that SLS is also induced upon depletion of TIMRHOM1, a factor involved in protein translocation into the mitochondria (28), supporting the role of PK3 as the kinase that regulates homeostasis between the ER and the mitochondria.

Two T. brucei iRhomboid proteins exist, TIMRHOM1 and TIMRHOM2, and were shown to be genuine components of the inner mitochondrial import system and to associate with a protein translocation intermediate stalled on its way into the mitochondria (28). However, the level of only TIMRHOM1 was elevated in SLS-induced cells (27). In mammals, iRhomboid proteins, especially from the DERLIN family, were shown to be associated with the ER and directly involved in ERAD (49). The fact that iRhomboid proteins lack proteolytic activity but can bind and stabilize unfolded domains makes them most suitable to regulate the fate of membrane proteins (50). However, it was recently suggested that the two trypanosome Rhomboid-like proteins are functional homologues of the yeast presequence translocation pore to the mitochondria. Of note, the trypanosome Rhomboid-like proteins are not related to the eukaryotic iRhomboid proteins (DERLIN and PARL) nor to their bacterial counterparts, and thus may have originated from the endosymbiont that gave rise to the mitochondrion (28).

The observation that PK3 is phosphorylated and trafficked upon depletion of ER factors and the mitochondrial TIMRHOM1 suggests that it may respond to perturbations taking place in either the ER or mitochondria. If so, it is possible that this kinase is localized to ER-mitochondrion contact sites. An ER-mitochondrion encounter structure (ERMES) was described and shown in yeast to be important for lipid transport and calcium signaling (51). In mammalian cells, the mitochondrion-ER contact sites (MERC) mediate the interaction between the mitochondrial porin voltage-dependent anion selective channel protein 1 (VDAC1) and the ER Ca^2+^ channel (inositol 1,4,5-trisphosphate receptor IP_3_R) (30). It is unclear at this time what type of ERMES exists in trypanosomes. A functional homologue of Mdm10 that is part of the yeast ERMES was identified in T. brucei and named TAC40. TAC40 mediates the linkage between mitochondrial DNA and the basal body connecting the mitochondrion with the flagellum of the parasite. However, no similar ERMES mediating the contact between the ER and the mitochondria and related to the yeast complex has been identified in trypanosomes (52). In addition, the Ca^2+^ channel IP_3_R, which is part of mammalian ER contact sites (30), localizes to trypanosome acidocalcisomes, which is the organelle mediating Ca^2+^ storage and bearing membrane contact sites with the mitochondria (53). Thus, we still do not know which trypanosome proteins mediate the contact between the ER and mitochondria. We are currently identifying the proteins associated with PK3 under normal conditions and upon SLS induction, hoping to identify proteins that contact PK3 and constitute the ER-mitochondrion contact sites. In mammalian cells, the PERK kinase controls mitochondrial function by attenuating mitochondrial protein import in response to stress, thereby preventing the accumulation of damaged or nonnative proteins that can interfere with mitochondrial function in response to ER stress (54). In addition, PERK induces upregulation of the level of stress-induced mitochondrial hyperfusion (SIMH), a protective mechanism that suppresses pathological mitochondrial fragmentation and promotes mitochondrial functions such as ATP production, which helps prevent mitochondrial fragmentation (55).

The finding that depletion of proteins involved in ER quality control does not induce SLS in PCF is intriguing and indicates that these factors may not be necessary for the survival of PCF trypanosomes. Indeed, stress-induced misfolding occurs along the life cycle of these parasites. The nondividing transmissive forms of the parasite, short “stumpies” (mammals to insect) and metacyclics (insect to mammals), may require ERAD capacity for cellular remodeling upon transmission. BSF ERAD is presumably critical because of antigenic variation. During antigenic variation, different versions of VSG can be formed, but many versions are likely to be misfolded. There must therefore be a system with “disposal capacity” to cope with the stress of misfolded VSG to be able to survive catastrophic switches. ERAD may provide the mechanism to cope with such unsuccessful switches (56). Indeed, ERAD was demonstrated in trypanosomes when a mutated version of transferrin receptor accumulated in the ER and was degraded by the proteasome (56). Thus, ERAD functions, and possibly all the factors that were studied here that did not induce SLS, such as UGGT, GLU2, and EDEM, are not essential in PCF grown under normal conditions. However, these factors were shown to be essential in BSF, supporting the crucial role of ERAD at this life stage (26), as stated above.

A recent study suggested that SLS induction is a stage-specific pathway, as *sec63* silencing did not induce SLS in BSF (57). However, the silencing in that study was done for a relatively short time (24 h), which is likely insufficient for generating defects in protein translocation necessary to induce SLS. The studies in PCF were always performed under longer silencing periods (2 to 3 days) (7, 9). We have previously shown that *sec63* silencing in BSF induces SLS after at least 2.5 days (7).

SLS is induced following treatment with compounds known to induce ER stress in other eukaryotes (7) or exposure to low pH (9). However, SLS is only induced following depletion of some essential factors (58). For example, SLS is not induced when depleting the SRP protein SRP54, despite being essential for viability (59). Depletion of SEC71, affecting the biogenesis of signal peptide (SP)-containing proteins but not polytopic membrane, also did not induce SLS (8). Note that silencing of factors involved in *trans*-splicing results in the accumulation of SL RNA and its secretion from the cell (58). Heat shock in both life stages of the parasite also induces an increase in the level of SL RNA and its secretion. SLS induction is a point of no return and represents a decision taken by the parasite to initiate a death pathway.

This study demonstrates that PK3, like other eIF-2α kinases, undergoes phosphorylation on multiple sites. To this end, we used PhosphoSitePlus database (http://www.phosphosite.org) (60) and compared the phosphorylation sites of human and mouse PERK with those observed on PK3 (see Fig. S2 in the supplemental material). To be able to evaluate the functional importance of the modified positions, we compared the kinase domain of PERK to the trypanosome homologues of eIF-2α (PK1 and -2) as previously described (41). We also compared all the reported phosphorylation sites on the different PERK kinases (50, 51, 61–76) to that of PK3 (Fig. S2). We noticed that Y554 of PK3 is analogous to the phosphorylated Y619 in the human kinase. Despite identifying 23 peptides covering this residue, there was no evidence of its phosphorylation under SLS activation. Failure to detect such a phosphorylation site does not imply that it does not exist. Phosphorylation of S707 and S708 is not conserved in other kinases and seems to be specific to PK3. The conserved residue T917, an analogue of T980 of mouse PERK that is present in the kinase activation loop and essential for eIF-2α phosphorylation, is of great interest (38). Unfortunately, there was little coverage of the protein in this domain in the two MS data sets, and we have no evidence of its phosphorylation. Notably, none of the single mutations we introduced completely abolished the autophosphorylation of PK3. However, the mutation PK3^F771L^ completely abolished the kinase activity.

10.1128/mBio.02602-21.2 FIG S2 Multiple-sequence alignment PK3 to PERK kinase. Amino acid sequences of human (Hs), mouse (Mm), Drosophila melanogaster (Dm), and Caenorhabditis elegans (Ce) PERK and T. brucei (Tb) PK1, -2, and -3 kinases were aligned using the T-COFFEE Multiple Sequence Alignment Server (http://tcoffee.crg.cat/). The scores for the alignment are designated: BAD (indicated either in purple or green), AVG (i.e., average [indicated in yellow]), and GOOD (indicated in pink). The domains are marked (I to V) as adopted from PROSITE (https://prosite.expasy.org/scanprosite/). The phosphorylated amino acid residues are indicated in red. The UniProt accession IDs of human HsPERK, MmPERK, DmPERK, CePERK, TbPK1, TbPK2, and TbPK3 are Q9NZJ5, Q7TQC8, Q9NIV1, Q19192, Q384V1, Q584D7, and Q583N6, respectively. Download FIG S2, TIF file, 1.0 MB. Copyright © 2021 Okalang et al. 2021 Okalang et al. https://creativecommons.org/licenses/by/4.0/ This content is distributed under the terms of the Creative Commons Attribution 4.0 International license.

As opposed to PERK, a transmembrane domain protein localized to the ER membrane, PK3 only associates with the ER and, upon activation, translocates to the nucleus. The translocation to the nucleus depends on its phosphorylation, since the translocation of a PK3 mutant defective in ATP binding was compromised. It is not currently known whether all the phosphorylation events on PK3 are autophosphorylations or if other kinase(s) can act on PK3. Phosphorylation on PK3 by another kinase may still depend on recognition of autophosphorylated PK3, which does not occur in the PK3^F771L^ mutant.

**FIG 7:**
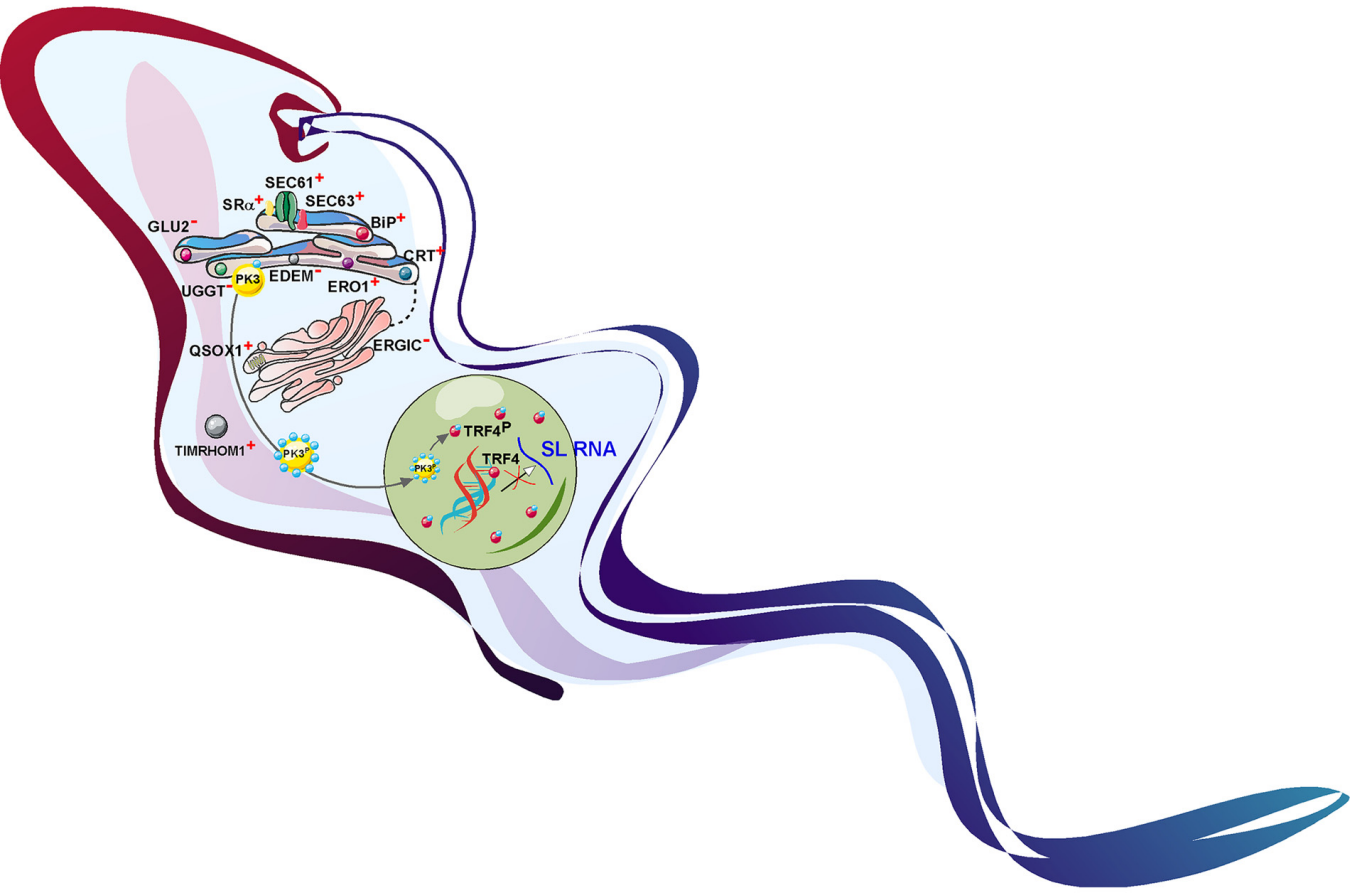
Protein factors whose depletion induces SLS and its mechanism. The scheme indicates the factors whose depletion induces SLS (marked with a plus sign [+]). Factors that do not induce SLS upon depletion are indicated with a minus (−) sign. During SLS, the PK3 undergoes phosphorylation on at least 9 sites. The PK3 migrates to the nucleus and phosphorylates TRF4, leading to detachment from its cognate promoter and resulting in cessation of SL RNA transcription.

A summary of the conditions or perturbations that induce SLS is presented in Fig. 7. This study provides evidence that SLS, which was initially discovered upon depletion of the SRP receptor, is activated by perturbation of a variety of factors involved in the translocation of proteins into the ER, their folding, and their modification along the intracellular protein trafficking pathway. In addition, SLS is also activated upon perturbations in the mitochondria. PK3 is therefore a key kinase in the cell, whose activation induces PCD, leading to parasite elimination. PK3 should therefore be considered an attractive drug target. A small molecule that would selectively activate PK3 could lead to suicide of these parasites.

## MATERIALS AND METHODS

### Cell growth and transfection.

Procyclic forms of T. brucei strain 29-13, which carries integrated genes for T7 polymerase and the tetracycline repressor (77), were grown in SDM-79 (78) supplemented with 10% fetal calf serum in the presence of 50 μg ml^−1^ hygromycin B and 15 μg ml^−1^ G418. Transfected cells were cloned by serial dilution to obtain a clonal population (77).

### Generation of transgenic parasites.

Listed in Table S1 in the supplemental material are the primers used to generate constructs for silencing the following genes: *bip* (Tb927.11.7460), *crt* (Tb927.4.5010), *glu2* (Tb927.10.13630), *uggt* (Tb927.3.4630), *ergic* (Tb927.11.4200), *edem* (Tb927.8.2910), *ero1* (Tb927.8.4890), *qsox* (Tb927.6.1850), and *timrhom1* (Tb927.9.8260). For most of the genes, we generated stem-loop constructs, but T7 opposing constructs were also used (77). Stem-loop RNAi constructs were linearized by EcoRV digestion, and T7 opposing constructs were linearized with NotI. The PTP-tagged proteins were prepared with PCR products amplified using primers listed in Table S1 and cloned into the PTP vector (79).

10.1128/mBio.02602-21.3 TABLE S1 List of primers used in this study. Download Table S1, DOCX file, 0.02 MB. Copyright © 2021 Okalang et al. 2021 Okalang et al. https://creativecommons.org/licenses/by/4.0/ This content is distributed under the terms of the Creative Commons Attribution 4.0 International license.

### PK3 construct preparation and site-directed mutagenesis.

The construct for tagging PK3 (Tb927.6.2980) with the C-terminal composite PTP tag was generated by cloning the PK3 gene fragment ∼1,600 nucleotides (nt) (amplified using the primers in Table S1) into the ApaI and NotI sites of pC-PTP-PURO (a kind gift from Laurie K. Read, University at Buffalo) (80), a derivative of pC-PTP-NEO in which the neomycin phosphotransferase coding region was replaced with that of puromycin *N*-acetyltransferase (79). The resultant PK3-PTP vector was amplified in Dam^−/−^ bacteria, linearized with BclI (NEB), transfected, and cloned into cells carrying the *sec63* stem-loop RNAi construct as previously described (8). The construct for tagging PK3 with the C-terminal MYC tag was generated as described above using primers listed in Table S1 into the HindIII and XbaI sites of pNAT plasmid carrying MYC tag (a kind gift from Sam Alsford, LSHTM) (81). Site-directed mutagenesis was performed using the primers listed in Table S1. The template plasmid was digested with DpnI (NEB), and clones were isolated. Mutations in the plasmids and isolated procyclic clones were verified by Sanger sequencing.

### Northern analysis and primer extension.

Total RNA was prepared with TRIzol reagent, and 20 μg/lane was separated on a 1.2% agarose gel containing 2.2 M formaldehyde. Small RNAs were fractionated on a 6% (wt/vol) polyacrylamide gel containing 7 M urea. Primer extension was performed as previously described (82, 83). The extension products were analyzed on a 10% denaturing polyacrylamide gel. RNA probes were prepared by *in vitro* transcription using [α-^32^P]UTP. Primers used for *in vitro* transcription are listed in Table S1.

### PK3 purification and mass spectrometry analysis.

Cells expressing the *sec63* silencing construct and PK3 PTP-tagged protein (27), uninduced or induced for 2.5 days, were grown to a density of 2 × 10^7^cells ml^−1^. The purification protocol used was essentially as described previously (79) from 10^10^ cells, but included the addition of phosphatase inhibitors to the extract (10 mM NaF, 1 mM sodium orthovanadate, and 50 mM β-glycerophosphate). In addition, the lysis buffer was modified to contain 0.5% deoxycholate, 0.5% NP-40, and 0.1% SDS to release the kinase from the membrane. After separation of purified proteins by denaturing 8% SDS-PAGE, protein bands were eluted from the gel and subjected to trypsin digestion. The resulting peptides were resolved by reverse-phase chromatography. Mass spectrometry was performed by an ion-trap mass spectrometer (Orbitrap XL; Thermo). To analyze phosphopeptides, the proteins were enriched on a TiO_2_ column. The mass spectrometry data were analyzed using the Discoverer software version 1.2 or 1.3 against the T. brucei TriTrypdb database (http://tritrypdb.org/tritrypdb/).

### Preparation of TRF4 and TIMRHOM1 antibodies and source of antibodies.

The *trf4* gene and a portion of the *timrhom1* gene were amplified by PCR using primers listed in Table S1. The amplified fragments were cloned into the pHIS vector (Novagen) and expressed in Escherichia coli BL21 cells. Recombinant proteins were extracted and purified using the Bugbuster reagent (Novagen, Inc.). To raise antibodies against TRF4 or TIMRHOM1, 400 μg of the proteins was emulsified with an equal volume of complete adjuvant (Difco). The emulsions were injected subcutaneously into two female New Zealand White rabbits. The first injection was followed by an additional two injections of 200 μg protein emulsified with an equal volume of incomplete adjuvant (Difco) at 2-week intervals. Serum was collected and examined for reactivity by immunofluorescence and Western analysis.

GRASP, SEC24, and GRIP70 antibodies were kindly provided by Graham Warren (Vienna University, Austria) (36, 84), antibody against *T. brucei* QSOX was prepared by the group of D. Fass (Weizmann Institute, Israel) using recombinant TbQSOX (85), PTB1 and ZC3H41 antibodies were prepared in our lab (58, 86), HSP83 antibody made against the *Leishmania* protein was kindly provided by Dan Zilberstein (Technion, Israel), and VH^+^-ppase antibody was kindly provided by Roberto Docampo (University of Georgia, USA). EP antibodies were purchased from Cedarlane, Canada. MYC (9E10) antibody was purchased from Santa-Cruz. The dilutions used are specified in the figure legends.

### Western analysis.

Whole-cell lysates (10^7^ cells) were fractionated by 10% SDS-PAGE, transferred to Protran membranes (Whatman), and reacted with antibodies. The bound antibodies were detected with goat anti-rabbit immunoglobulin G coupled to horseradish peroxidase and were visualized by ECL enhanced chemiluminescence (Amersham Biosciences).

### Immunofluorescence assay.

Cells were washed with phosphate-buffered saline (PBS), mounted on poly-l-lysine-coated slides, and fixed in 4% formaldehyde. Immunofluorescence was performed as described previously (9). Cells were visualized using a Nikon eclipse 90i microscope with Retiga 2000R (QImaging) camera.

### Phosphatidylserine exposure assay.

Trypanosomes were reacted with fluorescein isothiocyanate-labeled annexin V antibodies (MBL) and stained with propidium iodide (PI) according to the manufacturer’s instructions. The cells were analyzed by fluorescence-activated cell sorter (FACS) or visualized under a Zeiss LSM 510 META inverted microscope.

### PI staining for FACS analysis.

T. brucei cells were fixed in 70% ethanol–30% PBS and stored at 4°C overnight. Cells were then washed once with PBS and incubated on ice for 30 min to enable rehydration. The samples were resuspended in PBS containing 50 μg ml^−1^ RNase A (Roche Diagnostics) for 30 min at 4°C and stained with 50 μg ml^−1^ PI (Sigma). Samples and results were analyzed by FACS-Aria and FlowJo (FlowJo, LLC, Ashland, OR), respectively.
