# Supplementary figures and images for: The Spliced Leader RNA Silencing (SLS) Pathway in Trypanosoma brucei Is Induced by Perturbations of Endoplasmic Reticulum, Golgi Complex, or Mitochondrial Protein Factors: Functional Analysis of SLS-Inducing Kinase PK3
Source: mBio. 2021 Nov 30;12(6):e02602-21. doi: 10.1128/mBio.02602-21 (PMC8630539; doi:10.1128/mBio.02602-21)

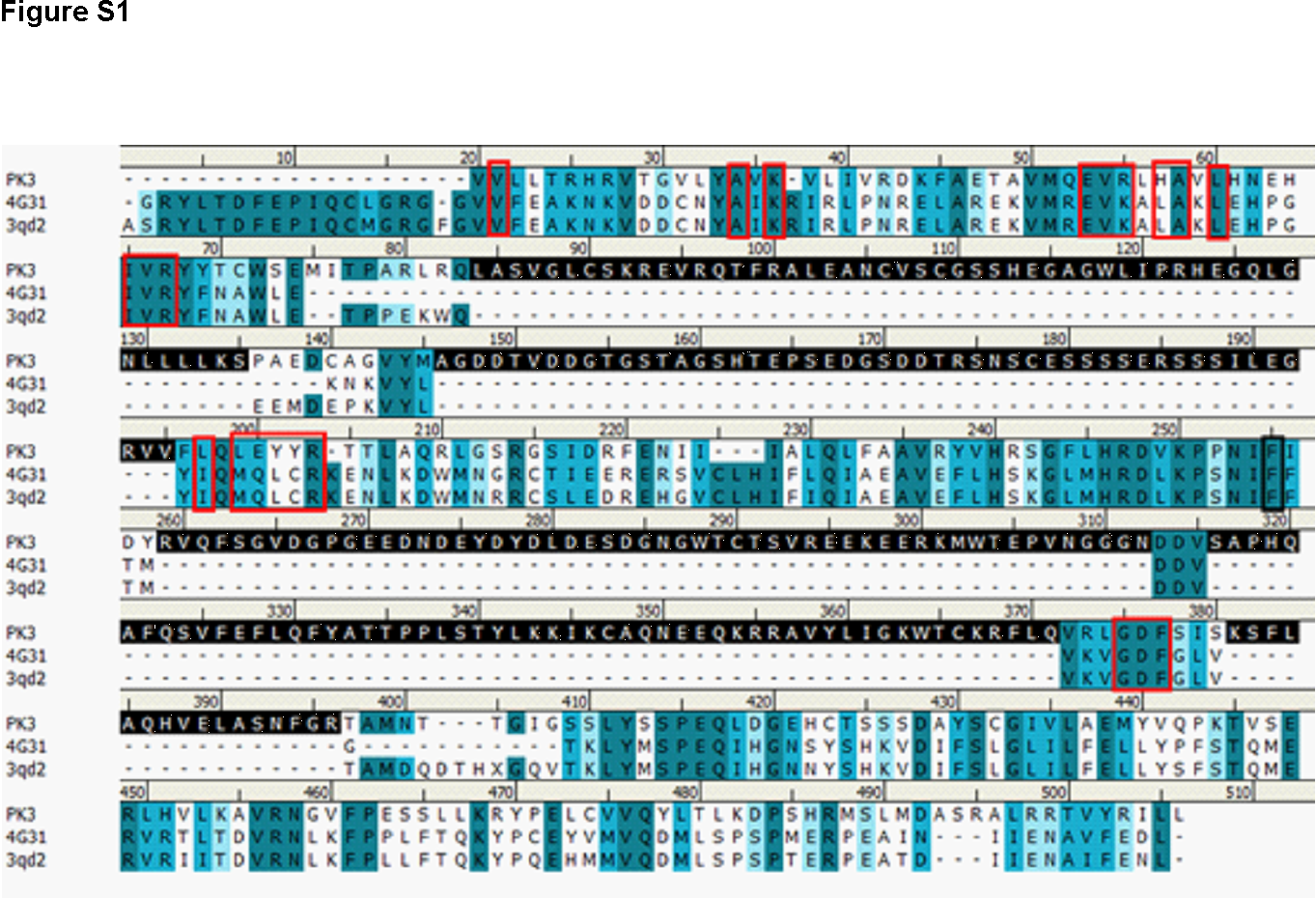

Supplement: FIG S1 [file mbio.02602-21-sf001.tif]

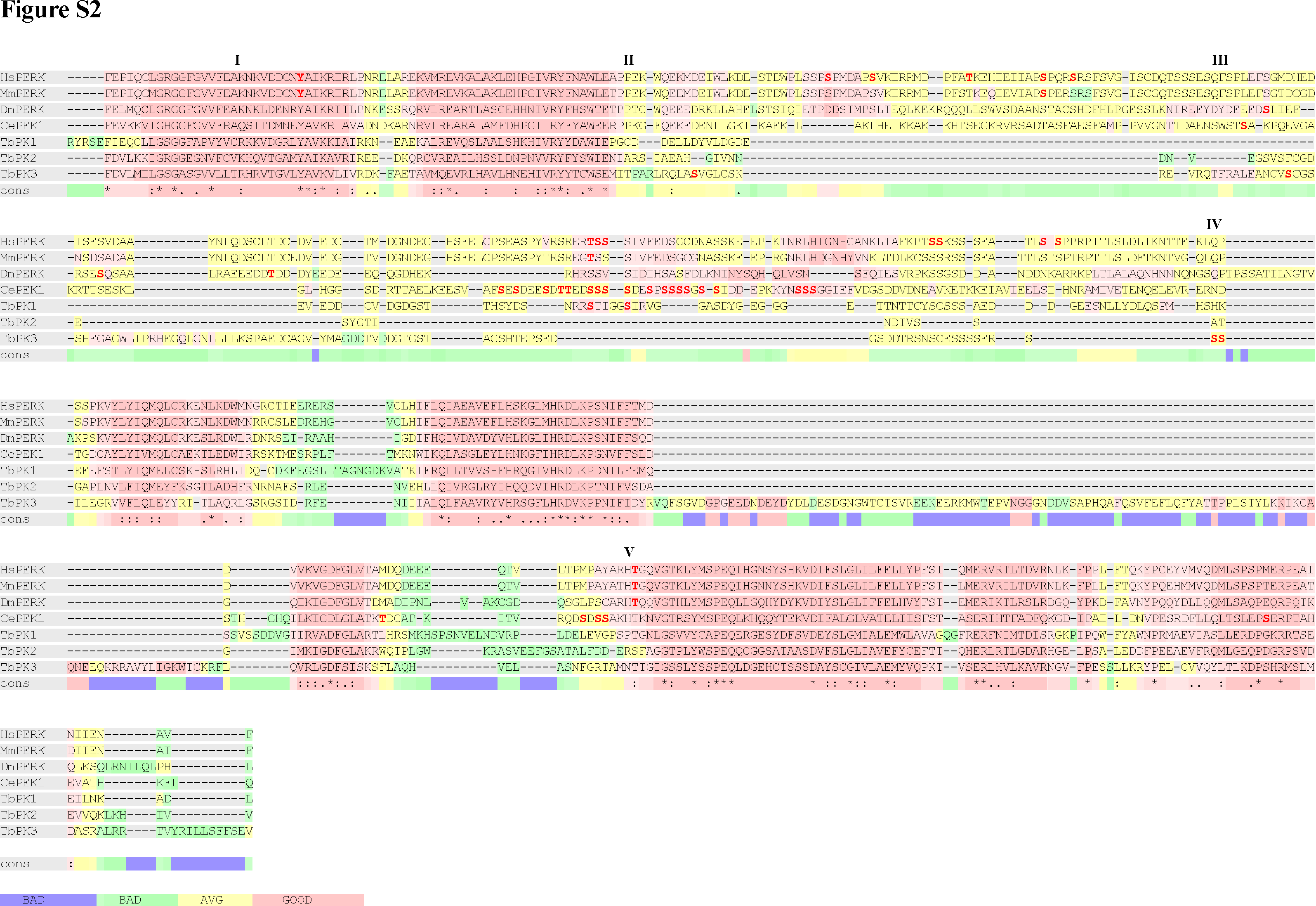

Supplement: FIG S2 [file mbio.02602-21-sf002.tif]
