# Supplementary material for: The Spliced Leader RNA Silencing (SLS) Pathway in Trypanosoma brucei Is Induced by Perturbations of Endoplasmic Reticulum, Golgi Complex, or Mitochondrial Protein Factors: Functional Analysis of SLS-Inducing Kinase PK3
Source: mBio. 2021 Nov 30;12(6):e02602-21. doi: 10.1128/mBio.02602-21 (PMC8630539; doi:10.1128/mBio.02602-21)
Supplement: TABLE S1 [file mbio.02602-21-st001.docx]

**Table S1. List of primers used in this study.**

| **Primers used for T7 opposing RNAi construct of *BiP* and *CRT*** | |
| --- | --- |
| **Identity** | **Sequence** |
| F_BIP_XhoI | 5’-TTTCTCGAGTTCAGCGAGAAAATCACA-3’ |
| R_BIP_HindIII | 5’-TTTAAGCTTGCGGTTATCCTTTGTCA-3’ |
| F_CRT_XhoI | 5’-TTTCTCGAGGGGAAACGAAGTATTGGCT-3’ |
| R_CRT_HindIII | 5’-TTTAAGCTTATGGACCCTTGTAAGCC-3’ |
| F_GLU2_XhoI | 5’-TTT CTC GAG GTA ACT ATC AAA GAT CCT-3’ |
| R_GLU2_HindIII | 5’-TTT AAG CTT TTT GCC ATG TTG TCT CC-3’ |
| F_UGGT_XhoI | 5’-TTT CTC GAG TGA GAA CAA AAC GTT T-3’ |
| R_UGGT_HindIII | 5’-TTT AAG CTT CCC TGC TTG GT-3’ |
| F_ERGIC_XhoI | 5’-TTT CTC GAG GGT GTT GGA GTA GTG TTC G-3’ |
| R_ERGIC_HindIII | 5’-TTT AAG CTT ATT TTC CTG CTC ATG AGT-3’ |
| F_EDEM_XhoI | 5’-TTTCTCGAGATGCATCCTATTCAAGCT-3’ |
| R_EDEM_HindIII | 5’-TTTAAGCTTCCATATGGTATCCCAGTG-3’ |
| **Primers used for stem-loop RNAi constructs** | |
| **Identity** | **Sequence** |
| F_GLU2_XbaI | 5’-TTTTCTAGAGGTAACTATCAAAGATCCT-3’ |
| R_GLU2_MluI | 5’-TTTACGCGTTTTGCCATGTTGTCTCC-3’ |
| R_GLU2_HindIII | 5’-TTTAAGCTTTTTGCCATGTTGTCTCC-3’ |
| F_EDEM_XbaI | 5’-TTTTCTAGAATGCATCCTATTCAAGCT-3’ |
| R_EDEM_MluI | 5’-TTTACGCGTCCATATGGTATCCCAGTG-3’ |
| R_EDEM_HindIII | 5’-TTTAAGCTTCCATATGGTATCCCAGTG-3’ |
| F_UGGT_XbaI | 5’-TTTTCTAGATGAGAACAAAACGTTT-3’ |
| R_UGGT_HindIII | 5’-TTTAAGCTTCTTCCATATGGTATCCCAGTG-3’ |
| R_UGGT_MluI | 5’-TTTACGCGTCTTCCATATGGTATCCCAGTG-3’ |
| F_ERGIC_XbaI | 5’-TTTTCTAGAGGTGTTGGAGTAGTGTTCG-3’ |
| R_ERGIC_MluI | 5’-TTTACGCGTATTTTCCTGCTCATGAGT-3’ |
| R_ERGIC_HindIII | 5’-TTTAAGCTTATTTTCCTGCTCATGAGT-3’ |
| F_iRhomboidI_XbaI | 5’-CCCTCTAGACTCGCCGATGCATTTAAT-3’ |
| R_iRhomboidI_MluI | 5’-TTTACGCGTCCGCATTTTCATGTGACA-3’ |
| R_iRhomboidI_HindIII | 5’-CCCAAGCTTCCGCATTTTCATGTGACA-3’ |
| **Primers used for C-terminal PTP tagging constructs and mutations** | |
| **Identity** | **Sequence** |
| F _BIP-PTP_ApaI | 5’-AAAGGGCCCGAGTCAGGTCTTTTCTAC-3’ |
| R _BIP-PTP_NotI | 5’-AAAGCGGCCGCCCCAGATCGTCCATGGGTT-3’ |
| F_CRT-PTP_ApaI | 5’-ATCAAACCCAAAGTCGAAG-3’ |

| R _CRT-PTP_NotI | 5’-AAAGCGGCCGCCCCAAGTCTGACTTATCCTC-3’ |
| --- | --- |
| F_GLU2-PTP _ApaI | 5’-AAA GGGCCC ATTCTTATGCCACTGGTG-3’ |
| R_GLU2-PTP_NotI | 5’-AAA GCGGCCGC CC TCTCTTCAGCACAATGG-3’ |
| F_EDEM-PTP_ApaI | 5’-AAAGGGCCCGGAATTTGGAGACATGGT-3’ |
| R_EDEM-PTP_NotI | 5’-AAA GCGGCCGC CC TCGAAACACAGACTTC-3’ |
| F_UGGT-PTP_ApaI | 5’-AAA GGGCCC CTAACGATGCCACAACAA-3’ |
| R_UGGT-PTP_NotI | 5’-AAA GCGGCCGC CC CATCATCCCACTTGAATCC-3’ |
| F_ERGIC-PTP_ApaI | 5’-AAA GGGCCC GGAGCACAAGGAAGAAGAG-3’ |
| R_ERGIC-PTP_NotI | 5’-AAA GCGGCCGC CC GTCACGACGATCTCTCGAG-3’ |
| F_PK3 _PTP_ApaI | 5’-AAAGGGCCCTGTCGAGCGGTGCGTTAGTGCTTGGGAAGA-3’ |
| R_PK3 _PTP _NotI | 5’-AAAGCGGCCGCCCCACCTCAGAAAAAAAGGAGAGCAATATACG-3’ |
| F_PK3_PTP_S606A | 5’-ACCAGCGCGGTTGCGCCAACTTGCCGCCGTTGGTCTTTGCAGCAAAAGGGAAG-  3’ |
| R_PK3_PTP_S606A | 5’-CTTCCCTTTTGCTGCAAAGACCAACGGCGGCAAGTTGGCGCAACCGCGCTGGT-  3’ |
| F_PK3_PTP_S628A | 5’-TAGGGCTTTGGAAGCCAATTGTGTTGCCTGTGGAAGCAGCCATGAGGGTGCCG-  3’ |
| R_PK3_PTP_S628A | 5’-CGGCACCCTCATGGCTGCTTCCACAGGCAACACAATTGGCTTCCAAAGCCCTA-  3’ |
| F_PK3_PTP_S707A | 5’-TGAATCATCGTCTTCAGAACGAGCCTCATCAATTCTTGAGGGGCGTGTTG-3’ |
| R_PK3_PTP_S707A | 5’-CAACACGCCCCTCAAGAATTGATGAGGCTCGTTCTGAAGACGATGATTCA-3’ |
| F_PK3_PTP_S708A | 5’-TGAATCATCGTCTTCAGAACGAAGTGCCTCAATTCTTGAGGGGCGTGTTGTGT-  3’ |
| R_PK3_PTP_S708A | 5’-ACACAACACGCCCCTCAAGAATTGAGGCACTTCGTTCTGAAGACGATGATTCA-  3’ |
| F_PK3_F771L_F | TCACAGGGATGTAAAACCTCCCAACATATTaATTGATTACCGTGTACAATTTTCAG GAGTA |
| R_PK3_F771L_R | TACTCCTGAAAATTGTACACGGTAATCAATtAATATGTTGGGAGGTTTTACATCCC TGTGA |
| **Primers used for TRF4 antibody preparation** | |
| **Identity** | **Sequence** |
| F_TBP_NheI | 5’-AAAGCTAGCATGGACAATGACTTCACTG-3’ |
| R_TBP_HindIII | 5’-AAAAAGCTTTTACCTCTTTGCGTACTG-3’ |
| **Primers used to generate T7 PCR template for *in vitro* transcription** | |
| **Identity** | **Sequence** |
| R_T7_GLU2 | 5’-TTAATACGACTCACTATAGGGAGATTTGCCATGTTGTCTCC-3’ |
| R_T7 -UGGT | 5’-TTA ATA CGA CTC ACT ATA GGG AGA AAG CTT CCC TGC TTG GT-3’ |
| R_T7_ERGICS | 5’-TTA ATA CGA CTC ACT ATA GGG AGA ATT TTC CTG CTC ATG AGT-3’ |
| R_T7_EDEM | 5’-TTAATACGACTCACTATAGGGAGACCATATGGTATCCCAGTG-3’ |
| F-BiP | 5’-GAACGTAGTGCGCATCAT-3’ |
| R-BiP | 5’-TTAATACGACTCACTATAGGGAGACAATCTCGTGGATGTCACT-3’ |
| F-CRT | 5’-GGGAAACGAAGTATTGGCT-3’ |
| R-CRT | 5'-TTAATACGACTCACTATAGGGAGAATGGACCCTTGTAAGCC-3' |
| R_SL RNA | 5'-TTAATACGACTCACTATAGGGAGAAAAAAAATAAAAAAAATA-3' |
| F_SL RNA | 5'-ACTAACGCTATTATTAGAACAGTTTCTGTACTAT-3' |
| R_7SL RNA | 5’-TTAATACGACTCACTATAGGGAGACCGCCTCGCGACGACACTTG-3’ |
| F_7SL RNA | 5’-CCGCTCGAGAGCCGGAGCGCATTGCTCTG-3’ |
| F_TIMRHOM1_MluI | 5’-GGGACGCGTCCGCATTTTCATGTGACA-3’ |
| R_TIMRHOM1_T7 | 5’-TTAATACGACTCACTATAGGGAGACACTGCTACAAAAAGTTCGA-3’ |
